# Haematological parameter among drug resistant tuberculosis patients in Ibadan

**DOI:** 10.4314/ahs.v24i1.3

**Published:** 2024-03

**Authors:** Pelumi Daniel Adewole, Tosin Deborah Ogundipe, Olumuyiwa Samuel Alabi, Abdulrazak Nuhu

**Affiliations:** 1 Department of Medical Laboratory Science, College of Health Sciences, Bowen University, Iwo, Osun State, Nigeria; 2 Department of Medical Laboratory Science, Faculty of Pure and Applied Science, Kwara State University, Malete, Kwara State, Nigeria; 3 Department of Pharmaceutical Microbiology, Faculty of Pharmacy, University of Ibadan, Oyo State, Nigeria

**Keywords:** Haematological parameters, drug resistant tuberculosis, Ibadan

## Abstract

**Background:**

Haematological abnormalities are common among tuberculosis patients but there is dearth of information on their value as prognostic markers in Multidrug resistant tuberculosis patients. This study examined the association between complete blood count variables and drug resistant tuberculosis.

**Materials and methods:**

Nighty (90) consenting adults comprising 30 Drug Resistant Tuberculosis patients (DR-TB), 30 Drug susceptible tuberculosis patients (DS-TB) and 30 healthy participants were recruited in this study. Ethical approval was obtained from Oyo State Ministry of Health Institutional Review Board while patients' demographic data were collected using structured questionnaire. Five milliliters (5mL) of blood samples were collected in EDTA bottle. Haematological parameters were analysed using impedance technique and Mindary-BG5380 5-part automated system.

**Result:**

The mean hemoglobin levels were significantly lower in DR-TB patients (11.70 ± 2.73 g/dL) than in DS-TB patients (8.33 ± 9.56 fL), with a mean difference of -3.37 ± 12.29 g/dL. The mean MCH and MCHC levels were also slightly lower in DR-TB patients (26.17 ± 3.44 pg and 30.41 ± 1.92 g/dL, respectively), but the differences were not statistically significant. The WBC count was similar in both groups (8.20 ± 3.80 × 10^^9^ /L and 8.45 ± 3.63 × 10^^9^ /L, respectively).

**Conclusion:**

The mean hemoglobin levels were significantly lower in DR-TB patients than in DS-TB patients which may be due to the increased inflammation associated with DR-TB. The WBC count was similar in both groups, suggesting that the immune system is responding similarly to the infection in both DR-TB and DS-TB patients.

**Recommendation:**

In the meantime, healthcare providers should be aware of these potential differences and use them to inform their diagnosis and treatment of patients with tuberculosis.

## Introduction

The emergence of drug resistant tuberculosis (DR-TB) has become a major public health obstacle in the treatment and control of tuberculosis globally^1^. Drug resistance in *M. tuberculosis* isolates which arises from spontaneous genetic mutation can be enhanced by poor adherence of the patients to the anti-TB drugs^2^. According to the World Health Organization (WHO), 20% of all reported TB cases worldwide are resistant to at least one anti-TB drug. In the year 2021, 450,000 cases of multidrug-resistant TB (MDR-TB) and 191,000 deaths were reported. MDR-TB is resistant to at least rifampicin and isoniazid, the two most effective TB drugs.^3^

Tuberculosis is a serious infectious disease that can affect the cellular elements of blood. For example, TB can cause anemia, which is a decrease in the number of red blood cells. This is because TB can damage the bone marrow, where red blood cells are made. TB can also cause decreased levels of white blood cells, which are important for fighting infection. Additionally, TB can cause increased levels of platelets, which are involved in blood clotting 4-5.

A full blood count is routinely requested for patients, regardless of the type of infection or disease, to provide detailed knowledge about diseases affecting the cells in the blood. These include: important information about red blood cells, white blood cells, haemoglobin estimation, hematocrit, RBC indices, white blood cell differentials, erythrocyte sedimentation rate, and red cell distribution width. These parameters provide the necessary information for decision-making at the initial stage of treatment 6-7.

However, there is a dearth of information on haematological parameters in TB patients. This study investigated haematological parameters among drug-resistant tuberculosis patients attending a referral chest hospital in Ibadan

## Materials and methods

### Ethical approval/informed consent

Ethical approval was obtained from Oyo State Ministry Health Institutional Review Board with reference number: AD 13/479/1539. At an individual level, inform consent was received from each of participants before sample and data collection.

### Research design

This was a prospective case-control study among the patient attending Jericho chest clinic hospital, the participants where group into 3 which are drug resistance tuberculosis patient, drug Susceptible tuberculosis and healthy individuals.

### Study population

The population in this study were tuberculosis patients diagnosed to be Anti-tuberculosis drug-resistant and drug-susceptible patients as well as healthy individuals who consented to participate in the study. Resistant Tuberculosis Patients, Drug Susceptible and healthy individual.

### Study duration

This study was conducted within the period of 9 months (March - November, 2020) from sample collection, processing and analysis.

### Sample size

A total of 90 samples were collected. This sample size was computed from previous study done by using the formular designed by Bolarinwa (2020) (8).


$$n=\frac{2(Z\alpha +Z\beta {)}^{2}\times \pi (1-\pi )}{(P1-P2)/2}$$

n = the minimum sample size

Z α Standard normal deviate at 80% of power set at 0.84, P1 = prevalence of DR-TB in Nigerian =2.2% 9. e = the level of error tolerance (10%), P2= the Prevalence rate of *M. tuberculosis* (50%) = 0.5.

### Sampling techniques

A purposive sampling technique was used in the selection of healthy individual who meet the inclusion criteria, while for the case TB patients who also meet the inclusion criteria were recruited on their visit to the TB clinic.

### Sputum and blood sample collection

Sputum samples were collected from patients into a screw capped, leak proof specimen containers. The samples were transported at 4°c into the laboratory and analysed within 24hours of collection. For each patient, 5mls of venous blood was collected into the EDTA bottle for patients found to be in the category of Drug Resistant Tuberculosis (DR-TB), Drug susceptible Tuberculosis (DST) and healthy subjects for the analysis of haematological parameter and Erythrocyte Sedimentation Rate.

### Laboratory investigations

For the determination of Haematological parameter, the Blood samples collected were analysed using Mindary BG5380 (5-part) haematological analyzer after dilution, aspiration and mixing before the determination for each parameter. The manufactural instructions were followed strictly.

### Data collection

The socio-demographic characteristics of respondents and TB history were obtained with the aid of structured questionnaire from each participant.

### Inclusion/exclusion criteria

Patients diagnosed to have tuberculosis and individuals confirmed by gene expert not to have TB and are ready to sign the informed consent form were enrolled in to the study. Those who decided otherwise were excluded from the study.

### Data analysis

Statistical Package for Social Sciences (SPSS) and descriptive statistics was used to analyses the data. Unpaired t-test was used for Statistical Comparison Calculation. The significance of difference in Haematological parameter with respect to DR-TB, DS-TB and control were assessed using the Pearson correlation coefficient, ANOVA, Regression analysis when correlating the Haematological parameter at P-value <0.05 which indicate Statistical significance.

## Results

### Relationship between haematological parameters and drug resistant tuberculosis

The result describes the association between various hematological parameters, specifically the HB (hemoglobin) and the red cell indices (MCH and MCHC), in different groups of patients. The statistical analysis revealed significant associations at a p-value less than 0.05. In the case of DR-TB (drug-resistant tuberculosis) patients, the mean HB was significantly lower (11.70 g/dL) compared to healthy patients (14.1 g/dL). Additionally, the red cell indices MCH and MCHC were also significantly lower in DR-TB patients (26.17 pg and 30.41 g/dL) compared to healthy individuals (29.41 pg and 34.20 g/dL, respectively) (Table 1).

**Table 1:** Comparison of Hematological parameter between MDR-TB and Healthy Control (Unpaired t-test)

Parameter	DR-TB Mean ±SD	Healthy Control Mean ±SD	P-Value
**RBC COUNTS (10^12^/L)**	4.69±1.34	5.22±0.93	0.08
**PCV (%)**	38.37±8.04	42.65±12.30	0.12
**HB(g/dL)**	11.70±2.73	14.1±3.14	0.02*
**RDW (%)**	16.54±2.35	16.61±1.77	0.10
**MCV(FL)**	86.25±11.49	91.30±13.32	0.90
**MCH (pg)**	26.17±3.44	29.41±5.22	0.01*
**MCHC(g/dL)**	30.41±1.92	34.20±3.15	0.01*

Note. Number of DR-TB= 30, number of Healthy Control=30, Total N=60

However, when comparing the hematological parameters between MDR-TB (multidrug-resistant tuberculosis) and DS-TB (drug-susceptible tuberculosis) patients, there was no statistical significance observed at a p-value less than 0.05(Table 2). In DS-TB patients, the mean values of MCV (mean value 83.30 FL), MCH (mean value 25.16 pg), and MCHC (mean value 30.25 g/dL) were significantly lower compared to the healthy control group (Table 3).

**Table 2:** Comparison of Hematological parameter between MDR-TB and DS-TB (Unpaired t-test)

Parameters	DR-TB Mean ± SD	DS-TB Mean ± SD	P-value
**RBC COUNTS (10^12^/L)**	4.69±1.34	4.88±1.20	0.57
**PCV (%)**	38.37±8.04	40.00±10.28	0.28
**HB(g/dL)**	11.70±2.73	12.29±3.44	0.47
**RDW (%)**	16.54±2.35	16.59± 8.64	0.98
**MCV(FL)**	86.25±11.49	83.30 ± 9.56	0.29
**MCH (pg)**	26.17±3.44	25.16 ± 2.49	0.19
**MCHC(g/dL)**	30.41±1.92	30.25± 1.77	0.74

Note. Number of DR-TB= 30, DS-TB =30, Total N=60

**Table 3:** Comparison of Hematological parameter between DS-TB and Healthy control (Unpaired t-test)

Parameters	DS-TB Mean ± SD	Healthy Control Mean ±SD	P-value
**RBC COUNTS (10^12^/L)**	4.88±1.20	5.22±0.93	0.22
**PCV (%)**	40.00±10.28	42.65±12.30	0.37
**HB(g/dL)**	12.29±3.44	14.1±3.14	0.08
**RDW (%)**	16.59± 8.64	16.61±1.77	0.99
**MCV(FL)**	83.30 ± 9.56	91.30±13.32	0.01*
**MCH (pg)**	25.16 ± 2.49	29.41±5.22	0.02*
**MCHC(g/dL)**	30.25± 1.77	34.20±3.15	0.01*

Number of DS-TB= 30, number of Healthy Control=30, Total N=60

Furthermore, the one-way ANOVA analysis demonstrated a statistically significant association of MCV, MCH, and MCHC when comparing all three groups (DR-TB, DS-TB, and Healthy control) (Table 4).

**Table 4:** Comparison of Haematological parameters between MDR-TB, DS-TB, healthy control (one – way Anova)

Parameters	DR-TB	DS-TB	Healthy control	F-value	P-value
**RBC COUNTS (10^12^/L)**	4.69±1.34	4.88±1.20	5.22±0.93	1.58	0.21
**PCV (%)**	38.37±8.04	40.00±10.28	42.65±12.30	1.31	0.28
**HB(g/dL)**	11.70±2.73	12.29±3.44	14.1±3.14	4.83	0.01*
**RDW (%)**	16.54±2.35	16.59±8.64	16.61± 1.77	0.01	0.99
**MCV(FL)**	86.25±11.49	83.30±9.56	91.30± 13.32	3.68	0.03*
**MCH (pg)**	26.17±3.44	25.16±2.49	29.41± 5.22	9.80	0.01*
**MCHC(g/dL)**	30.41±1.92	30.25±1.77	34.20± 3.15	26.87	0.01*

Note. Number of DR-TB= 30, number of DS-TB=30, number of Healthy Control=30, Total N=90

### Relationship between haematological parameters and drug resistant tuberculosis

The mean value of WBC count was significantly increase in the DR-TB Patients (8.2× 10^^^9 /L) than in the controls (5.42 × 10^^^9 /L) (Table 5). When comparing the haematological parameters among the MDR-TB and DS-TB patient it shows no statistically significant association at P-value < 0.05 as shown on the table (6). In DS-TB, the mean value of these parameters when compared with DS-TB and Healthy controls, WBC with mean value of 8.45 × 109 was significantly increased than in Healthy group (5.42 x 109) at p-value < 0.05 as shown on (Table S7). Using one-way Anova, there was a statistically significant association of WBC when comparing the three groups (DR-TB, DS-TB and Healthy control as shown on the (TableS8).

**Table 5:** Comparison of Hematological parameter between MDR-TB and Healthy Control (Unpaired t-test)

Parameter	DR-TB Mean ±SD	Healthy control Mean ±SD	P-value
**WBC COUNTS (10^9^/L)**	8.20±3.80	55.80±16.40	0.01*
**NEUTROPHIL (%)**	59.46±17.24	55.80±16.40	0.40
**BASOPHIL (%)**	0.55±0.41	0.60±0.54	0.69
**EOSINOPHIL (%)**	3.13±4.09	3.52± 3.01	0.60
**MONOCYTE (%)**	4.33±3.67	4.07±2.11	0.74
**LYMPHOCYTES (%)**	32.54±37.68	36.13±20.10	0.46

Note. Number of DR-TB= 30, number of Healthy Control=30, Total N=60

**Table 6:** Comparison of Hematological parameter between MDR-TB and DS-TB (Unpaired t-test)

Parameter	DR-TB Mean ±SD	DS-TB Mean ± SD	P-value
**WBCCOUNTS (10^9^/L)**	8.20±3.80	8.45 ± 3.63	0.79
**NEUTROPHIL (%)**	59.46±17.24	61.44 ± 15.02	0.64
**BASOPHIL (%)**	0.55±0.41	0.54 ± 0.34	0.92
**EOSINOPHIL (%)**	3.13±4.09	3.52± 3.01	0.68
**MONOCYTE (%)**	4.33±3.67	4.16± 3.54	0.86
**LYMPHOCYTES (%)**	32.54±37.68	30.34 ±14.97	0.59

Note. Number of DR-TB= 30, DS-TB=30, Total N=60

## Discussion

Tuberculosis continues to be an important communicable disease in the world and is a major public health problem in Nigeria. Haematological abnormality is a common finding among Tuberculosis patients 9. Although, haematological parameters have been routinely tested in clinical laboratory using automated Haematological analyzer hitherto, there is dearth of information on their value as prognostic markers in Multidrug resistant tuberculosis patients.

In this present study, Hb, MCH and MCHC were significantly reduced among DR-TB when compare to Healthy control and no significance change in these RBC count, PCV, MCV, MCH, MCHC and RDW among MDR-TB when compare to DS-TB. This is consistence with previous work done in India by Gowda *et al.* (2017) and Arulselvan *et al.* (2017) who found that patients with DR-TB had significantly lower hemoglobin levels than patients with DS-TB 4,10. Similarly, the lack of significant changes in RBC, PCV, MCV, and RDW among patents with MDR-TB compared to those with DS-TB suggests that the anemia in MDR-TB patients is not due to changes in the size or shape of red blood cells, but rather to a decrease in the total number of red blood cells 11.

Furthermore, only WBC counts were significantly increased among DR-TB when compare to healthy control, and no significance change in Neutrophils, Lymphocytes, Monocytes, Basophils and Eosinophils among DR-TB when compared to Healthy individuals. This is similar to study carried out in Turkey by Ursavas et al., (2010) 12. They reported that WBC counts showed MDR-TB patients were closed to having leucocytosis. The main reason for the change in blood leukocyte concentration can be due to indication of frequent continuous inflammatory response 12,14 Contrarily, the study done in India by Arulselvan *et al.* (2022) 13 revealed that patients with DR-TB had significantly lower lymphocyte counts and higher neutrophil counts than patients with drug-susceptible tuberculosis (DS-TB).

### Strengths of the study

This study was able to determine the specific haematological parameters associated with drug-resistant tuberculosis in Ibadan, Nigeria.

## Conclusion

This study highlights the significant difference in haemoglobin levels between DR-TB and DS-TB patients, indicating lower hemoglobin levels in the former group. Additionally, it demonstrates a higher ESR in DR-TB patients, suggesting increased inflammation or infection. These findings contribute to the growing body of literature on the hematological profile of DR-TB patients and provide valuable insights for clinicians and researchers in managing and understanding the disease.

## Recommendations

Haematological parameters tests should be done routinely for MDR-TB patients. Future research that will include lager sample size on MDR-TB and DS-TB patients should be carried out to see the effect of the anti-tuberculosis drugs on the patients from time to time.
